# Chemokines and Cancer: Friends or Foes?

**Published:** 2020-06-29

**Authors:** Marianne Strazza, Adam Mor

**Affiliations:** Columbia Center for Translational Immunology, Columbia University Medical Center, New York, USA

**Keywords:** Immune checkpoint, T cells, Chemokines, Tumor infiltration, Cell trafficking

## Abstract

Immune cell infiltration into tumors, intratumoral cellular organization, and the cell-specific expression patterns of chemokines and chemokine receptors greatly influence the efficacy of immunotherapeutic treatment strategies. In our recent review article, we shined a light on the deciding role of the chemokine network between immune mediated tumor regression or immune evasion of the tumor. Current T cell centric immunotherapeutic strategies primarily rely on increasing cellular activation and decreasing cellular inhibition, with the overall goal of enhancing effector cell function. These strategies neglect to account for the presence of the T cells within the tumor, hardly boosting immune cell infiltration. Chemokines and chemokine receptors are the regulators of recruitment, migration, and intratumoral compartmentalization. Yet, utilizing the chemokine network to recruit immune cells that will drive tumor regression is not a straightforward path, as tumor cells often hijack these pathways in the effort of immune evasion. Many novel therapeutic strategies involving chemokine targeting are under trial for many diverse tumor types. As a field, we can learn from both the successes and failures of these trials in order to push forward the next generation of immunotherapeutic strategies that include augmented T cell trafficking.

## Introduction

The efficacy of checkpoint inhibitors in the treatment of cancer is linked to the degree of T cell infiltration in the tumor, specifically, the intratumoral localization of effector T cells relative to tumor cells, dendritic cells, and immune suppressive cell populations [1]. Chemokines regulate not only the movement of cells into and out of the tumor, but also the cellular organization within the tumor. Distinct “neighborhoods” are established within the tumor based on expression patterns of chemokines and chemokine receptors. Many tumors hijack the chemokine network in order to form a highly immunosuppressive environment. In these instances, suppressive cell populations are recruited into the tumor to establish a microenvironment through cytokine and chemokine secretion that is unsupportive of effector cell activation. We recently reviewed this interplay between immune cells, the tumor, and the chemokine network with a focus on the critical role played by chemokines in immune mediated tumor regression [2].

## Review of Literature

These considerations collectively contribute to the subtyping of the immune presence in tumors as either immune desert, excluded, or inflamed. Analysis of tumor histology can determine which of these three subtypes a tumor can be categorized as, allowing for a more customized immunotherapeutic approach that target the specific mechanisms that prevent the antitumor response. Immune desert tumors have little-to-no immune infiltration. Immune deserts indicate a lack of pre-existing anti-tumor immunity, and patients with these tumors would benefit from the induction of tumor-specific T cells [3]. The excluded subtype tumors are defined by the restriction of T cells to the stroma, and these patients will benefit from a therapy strategy that promotes the infiltration of T cells into the tumor parenchyma. The final tumor subtype, inflamed tumors, have relatively high levels of T cell infiltration in the tumor, however these T cells are not functioning properly. Inflamed tumors would most easily be targeted by immune checkpoint inhibitors. Limiting the growth of each of these tumor subsets can be better achieved by incorporating chemokine modulation therapy, though perhaps most apparently immune deserts and excluded tumors.

Despite the nuanced role of chemokines, intervention into this network is highly promising in combination with checkpoint inhibitors. In recent years, multitudes of clinical trials have included chemokine interventions. These trials can be generalized into two categories:

Neutralizing chemokines that will enhance immunosuppression, and
Incorporating chemokine receptors into T cell adoptive transfers to direct T cell trafficking post transfer.

### Targeting chemokines to curb immune suppression

Recruitment of myeloid-derived suppressor cells (MDSCs) into the tumor is a major mechanism of immune evasion by inhibiting T cell function by expressing arginase and inducible nitric oxide synthase and secreting immunosuppressive cytokines, including transforming growth factor-β (TGF-β) and IL-10 [4]. The presence of MDSCs in the periphery or within the tumor correlates with a lack of efficacy of PD-1 therapeutic interventions [4], and therefore, the combined targeting of MDSCs with checkpoint inhibitors can improve patient survival. There are a number of therapeutic strategies that can be employed to interfere with MDSCs function including blocking development and differentiation, inhibiting function, and limiting recruitment [5]. MDSCs are responsive to CCL2, CCL5, CCL7, CXCL1, CXCL5, and CXCL12, depending on subset [5–7]. The accumulation of MDSCs in human ovarian and gastric cancer microenvironments has been attributed to the expression of CXCR4 on MDSCs and the upregulation of CXCL12 within the tumors [5,8]. In a mouse glioma model, the administration of anti-CXCR4 antibody decreased tumor infiltration of MDSCs and in combination with anti-PD-1 antibody improved overall survival [5,9]. Building on this finding, a still ongoing Phase II clinical trial of the CXCR4 antagonist BL-8040 in combination with the anti-PD-1 antibody pembrolizumab in the treatment of pancreatic ductal adenocarcinoma (NCT02826486) found that objective response rate, overall survival, and disease control rate were all improved in patients that received the combined drug regimen [10]. Perhaps lending insight into the mechanism of this outcome, it was noted that BL-8040 increased CD8+ effector T cell tumor infiltration, decreased MDSCs, and decreased circulating regulatory T cells [10].

In the sera of colorectal cancer patients there is a noted increase in CCL15 and a striking intratumoral presence of MDSCs expressing its receptor CCR1 [5]. To explore the causative mechanism connecting these observations with tumor growth, CCL15 was genetically deleted from a human colorectal cancer cell line and implanted in an orthotopicxeno graft model. In this study it was concluded that CCL15 deletion was associated with diminished CCR1+ cell accumulation in the tumor and limited tumor growth [11]. Interestingly, analysis of 333 clinical specimens of primary colorectal cancer showed that CCL15 was expressed mainly at the invasion front, rather than the center of the tumor. This suggests that MDSCs may form a cellular barrier of immunosuppression, preventing T cell infiltration and function at the tumor boundaries. This again calls to mind the importance of cellular organization within the tumor microenvironment.

The CXCR1 and CXCR2 axes, including the ligands CXCL1, CXCL2, CXCL5, and CXCL8, also contribute to migration and recruitment of MDSCs [5]. In particular, CXCL8 which binds both CXCR1 and CXCR2 has been heavily implicated in malignant melanoma tumor progression [12–16]. Together this suggests that CXCR1 and CXCR2 inhibition is an attractive intervention strategy for malignant melanoma. A currently recruiting Phase I clinical trial (NCT03161431) will treat participants with melanoma for 21 days with SX-682, a CXCR1/2 inhibitor, as a monotherapy - then with pembrolizumab, an FDA approved immunotherapy for melanoma. All participants will be monitored for 3 months. After the monotherapy stage of the trial is complete, the next participants will receive SX-682 and pembrolizumab concurrently. These combination therapy participants will be evaluated for 2 years. SX-682 is an orally available allosteric inhibitor of CXCR1 and CXCR2 that has shown promise in promoting tumor regression pre-clinically. Specifically, SX-682 administration decreased MDSCs infiltration, increased T cell infiltration, enhanced T cell activation and cytotoxicity, promoted responsiveness to PD-1 blockade, limited tumor growth, and enhanced overall survival in mouse models of squamous cell carcinomas4. SX-682 holds much promise for success because of these diverse processes impacted by these pathways.

The recruitment of regulatory T cells (Tregs) into tumors also contributes to inhibition of effector T cell function. High levels of expression of CCR4 on Tregs lends rationale to the use of the anti-CCR4 antibody, mogamulizumab, in the treatment of solid tumors. Two Phase I trials combined mogamulizumab with the anti-PD-1 antibody nivolumab in the treatment of various solid tumors (NCT02946671 and NCT02476123). The published report from one of these trials notes a decrease in intratumoralTregs and an increase in intratumoral CD8+ T cells [17]. Though it is still early, the combination of these two antibodies has a lot of potential for promoting tumor regression.

### Enhancing the success of adoptive T cell transfer through chemokines

In the context of adoptive T cell transfer, chemokines can influence the recruitment of the T cells to the tumor and the intratumoral localization following infiltration. In prior discussions, much attention has been given to enhancing T cell recruitment. No less important, though, is the role of chemokines in properly localizing transferred T cells within the tumor and in supporting a microenvironment in which T cells can function. It has been previously reported that CD8+ T cells that are adoptively transferred into tumor bearing mice, rely on CXCR3 for tumor entry [18]. Additionally, following dual PD-1 and CTLA-4 blockade, the noted increase in CD8+ T cell infiltration was reduced with the addition of a neutralizing antibody for CXCR3 [19]. In another study, CXCR3 expression on CD8+ T cells was found to be essential for an anti-PD-1-induced anti-tumor response through a mechanism of co-localization of the T cells with dendritic cells inside the tumor [1]. Intratumoral interferon gamma (IFN-γ) is one proposed method to increase local expression of CXCR3 ligands CXCL9, 10, and 11. Collectively, there is an abundance of evidence for the necessary role of CXCR3 in an effective T cell response to tumors, and additional studies of modulation of CXCR3 and its ligands are warranted.

The CXCR1 and CXCR2 inhibitor SX-682 has already proven to promote tumor clearance following adoptive cell transfer of tumor antigen specific T cells in mouse models of squamous cell carcinomas [4]. In these studies, it is likely that the enhanced T cell function is secondary to the inhibition of MDSCs in the tumor. These receptors are also proving to be key players in novel iterations of chimeric antigen receptor (CAR) T cells. Expression of CXCL8, a ligand for these two receptors, has been shown to be markedly high in a number of different types of tumors and is increased further following radiation [20]. This observation led to the design of CAR T cells expressing CXCR1 or CXCR2, that demonstrate enhanced migration to and persistence in the tumor, which correlates with complete tumor regression and long-lasting immunologic memory in models of numerous aggressive tumors.

## Conclusion

The findings highlighted here teach an important lesson - in the conception of the next generation of immunotherapeutic strategies we cannot label chemokine axes as “helpful” or “harmful” in a binary manner. In moving forward, we are faced with the great challenge of appreciating chemokine axes for orchestrating a complex interplay of cell types. In the example of CXCR1 and CXCR2, it is evident that the chemokine ligands of these receptors are notably high in a number of tumors. This is initially advantageous to the tumor as MDSCs are recruited to support an immune suppressive environment. One strategy to combat this is to inhibit the function of these receptors on MDSCs to prevent recruitment. Another strategy relies on taking back these chemokine pathways by overexpressing these receptors in CAR T cells armed to fight tumor growth. The strategies may not need to be mutually exclusive, in fact the most promise for success appears to be in a combined approach. The neutralization of chemokine pathways in tumor and immune suppressor cells, the overexpression of chemokine receptors on T cells engineered for adoptive transfer, and the administration of immune checkpoint inhibitors should be considered complementary tactics.
